# Neuroprotection vs. Neurotoxicity: The Dual Impact of Brain Lipids in Depression

**DOI:** 10.3390/ijms26062722

**Published:** 2025-03-18

**Authors:** Yuting Yan, Yan Zhang, Mengting Liu, Lingjie Li, Yanrong Zheng

**Affiliations:** Zhejiang Key Laboratory of Neuropsychopharmacology, School of Pharmaceutical Sciences, Jinhua Academy, Zhejiang Chinese Medical University, Hangzhou 310053, China

**Keywords:** depression, lipid, sphingolipids, glycerophospholipids, fatty acyls, sterol lipids

## Abstract

Growing neurochemical evidence highlights cerebral lipid dysregulation as a key factor in the pathophysiology of major depressive disorder (MDD). This review systematically explores the dual roles of lipid species in both normal behavioral regulation and MDD development. By critically examining the recent literature, we classify these lipid species into two functional categories based on their functional neuroactivity: (1) neuroprotective lipids (sphingomyelin, cholesterol, cardiolipin, sphingosine, phosphatidic acid, and phosphatidylserine), which exert neuroprotective effects by modulating membrane fluidity and supporting synaptic vesicle trafficking; and (2) neurotoxic lipids (ceramides, phosphatidylinositol, phosphocholine, and phosphatidylethanolamine), which promote apoptotic signaling cascades and disrupt mitochondrial bioenergetics. An unresolved but critical question pertains to the maintenance of homeostatic equilibrium between these opposing lipid classes. This balance is essential, given their significant impact on membrane protein localization and function, monoaminergic neurotransmitter metabolism, energy homeostasis, and redox balance in neural circuits involved in mood regulation. This emerging framework positions cerebral lipidomics as a promising avenue for identifying novel therapeutic targets and developing biomarker-based diagnostic approaches for MDD treatment.

## 1. Introduction

Major depressive disorder (MDD) is a prevalent, heterogeneous, and recurrent neuropsychiatric condition, characterized by a wide range of symptoms, including mood disturbances and cognitive impairments. Its multifactorial etiology results from the complex interaction between environmental and genetic factors, and it often coexists with other medical or psychiatric conditions. While contemporary pharmacotherapy is effective for many patients, it has notable limitations, such as high rates of partial or non-response and a delayed onset of therapeutic effects. Recent studies have demonstrated that a single dose of ketamine can rapidly alleviate depressive symptoms in MDD patients, an effect that typically requires extended treatment with conventional antidepressants like fluoxetine [1,2]. An increasing body of preclinical and clinical research supports ketamine’s rapid and sustained antidepressant effects. However, the known risks of abuse and toxicity associated with long-term ketamine use [3,4] highlight the urgent need for novel pharmacological agents that can deliver rapid antidepressant effects without these adverse side effects [5].

Lipids are essential molecules that play crucial structural and functional roles within cells. The brain, which contains the second highest lipid content after adipose tissue, is rich in these vital molecules. Disruptions in lipid profiles within the central nervous system (CNS) have been linked to several disorders, including anxiety disorders, schizophrenia, and MDD [6]. Alterations in brain lipid metabolism have been shown in clinical responses to antidepressant therapy in patients with treatment-resistant depression [7]. Additionally, dysfunction in brain regions such as the cortex, hippocampus, striatum, and cerebellum, which are involved in various aspects of behavioral regulation and performance, has been associated with depression [8,9]. Given this, our review will focus on lipid composition changes across different brain regions in MDD and examine the dual roles of lipids in both neuroprotection and neurotoxicity.

## 2. Application of Various Lipids in MDD

Brain lipids are essential for numerous cellular functions, including the myelination of neuronal projections, neurotransmission, neural plasticity, energy metabolism, and neuroinflammation. They are crucial for determining the localization and function of proteins within neuronal membranes. Specifically, the structural integrity of cell membranes and the signaling processes they facilitate depend on maintaining lipid homeostasis in the brain. Lipids can also act as neurotransmitters or other signaling molecules. The diverse array of brain lipids supports a wide range of vital processes, and disruptions in lipid balance can cause significant damage to the central nervous system [10]. Recently, further progress has been made in the study of brain lipids. The authors of one article present a lipidome map of the human brain comprising 75 regions, showing lipidome patterns within the human brain, the association between the brain lipidome and the functional architecture [11].

**Figure 1:**
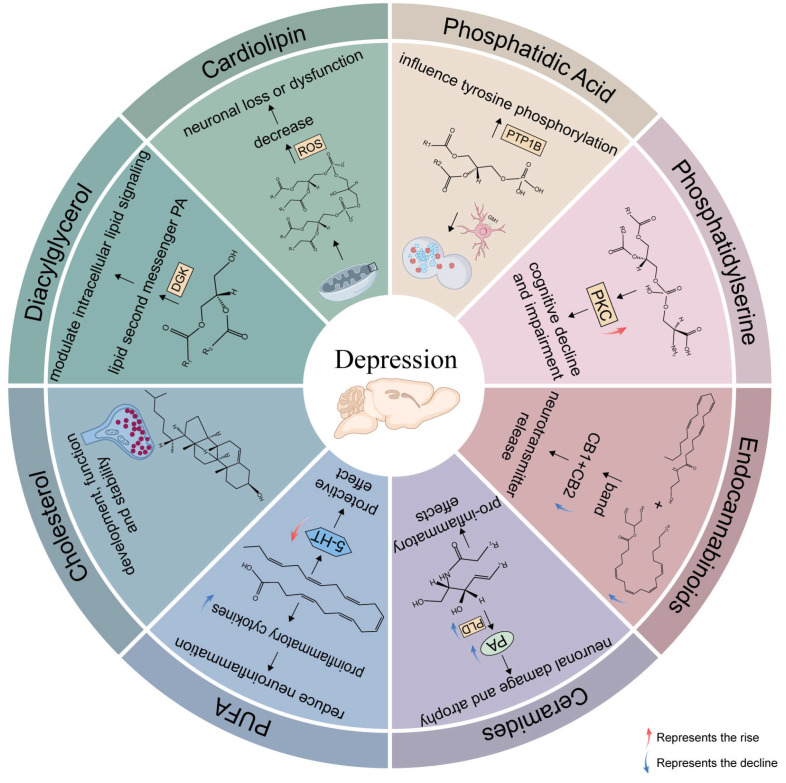
The role of major lipids in the brain after depression. An overview of the role of neuroprotective and neurotoxic lipids after depression. Phosphatidic acid (PA) mainly affects autophagy through GM1 on neurons and through PTP1B influences tyrosine phosphorylation. Phosphatidylserine (PS) mainly leads to cognitive decline through activating PKC. Anandamide and 2-arachidonic acid glycerol exert their effects primarily by binding to CB1 and CB2 receptors, and influence neurotransmitter release at presyn-aptic terminals. Ceramides cause neurological damage and pathology through the acid sphingomyelinase–ceramide pathway and have pro inflammatory effects. PUFA plays an activity protective effect by enhancing 5-HT and reduces the production of pro-inflammatory cytokines. Cholesterol affects synapses in development, function, and stability. Diacylglycerol affects lipid second-messenger PA through DGK and modulates intracellular lipid signaling. Cardiolipin reduces after depression, leading to neuronal loss or dysfunction. DGK, diacylglycerol kinase; ROS, reactive oxygen species; PTP1B, Protein Tyrosine Phosphatase-1B; PKC, protein kinase C; PA, phosphatidic acid; PLD, phospholipase D; 5-HT, 5-hydroxy tryptamine; CB1, type-1 cannabinoid receptor; CB2, type-2 cannabinoid receptor.

In 2005, the International Committee on the Classification and Nomenclature of Lipids (ICCNL) identified eight distinct categories of lipids, which are extensively documented in the LIPID MAPS Structure Database, and proposed changes and updates to the classification, nomenclature, and structural representation in 2009 [12]. The LIPID MAPS classification system is based on two core building blocks: ketoacyl groups and isoprene groups. Lipids are defined as hydrophobic or amphipathic molecules that can form through two types of condensation reactions: one involving carbanions of ketoacyl thioethers and the other involving carbocations of isoprene units. This system categorizes lipids into eight groups: fatty acyls, glycolipids, glycerophospholipids, sphingolipids, saccharolipids, polyketides, sterol lipids, and prenol lipids. We will explore each of these categories in detail, drawing on evidence from both clinical and animal studies (Figure 1).

### 2.1. Sphingolipids, SPs

Sphingolipids (SPs) are a class of amphipathic lipids that play essential roles in cellular membrane structure and function, particularly in the CNS. This lipid family includes ceramide, sphingosine, and sphingomyelin, which regulate key cellular processes, such as proliferation, differentiation, apoptosis, migration, and cell–cell interactions [11]. In the CNS, sphingolipids also function as critical signaling molecules. Bioactive sphingolipids, including ceramide, ceramide-1-phosphate (C1P), sphingosine, and sphingosine-1-phosphate (S1P), influence a wide range of biological functions, such as cell growth, adhesion, migration, inflammation, angiogenesis, and programmed cell death.

#### 2.1.1. Neurotoxic (Cer)

Ceramides, a major class of sphingolipids, consist of a sphingoid base linked to a fatty acid via an amide bond. They serve as key intermediates in the biosynthesis of complex sphingolipids, including sphingomyelins, cerebrosides, and gangliosides.

In recent years, the acid sphingomyelinase (ASM)–ceramide pathway has emerged as a critical contributor to depression pathophysiology. Increased ASM activity elevates ceramide levels in hippocampal endothelial cells, which subsequently inhibit phospholipase D (PLD), leading to decreased phosphatidic acid levels and exacerbating inflammation and apoptosis. These molecular changes impair neuronal proliferation, maturation, and survival, particularly in the hippocampus and prefrontal cortex (PFC), contributing to MDD [13,14,15]. Additionally, phosphorylation of ceramide to 1-phosphoceramide may activate phospholipase A2, a process also linked to MDD. Beyond their role in neuropsychiatric disorders, ceramides contribute to cardiovascular disease, enhance oxidative stress, and exert pro-inflammatory effects by modulating cytokine activity [13].

Several studies have shown that chronic stress increases ceramide (Cer) levels in the PFC and hippocampus [14]. However, no significant changes have been observed in the amygdala or cerebellum [16]. Specific ceramide species, such as Cer(22:1) and Cer(26:1), are elevated during chronic unpredictable stress (CUS), while other species, including Cer(16:0), Cer(16:1), and Cer(18:1), increase in the hippocampus and PFC [16]. Additionally, long-term corticosterone (CORT) exposure raises the levels of Cer(20:0), Cer(24:1), Cer(26:0), and Cer(26:1) in the ventral hippocampus, along with HexCer(16:0), HexCer(18:0), HexCer(18:1), HexCer(20:0), HexCer(26:0), HexCer(26:1), LacCer(20:0), and LacCer(26:1) in the dorsal hippocampus [17]. In the chronic variable stress (CVS) model, Cer(m44:3) levels were elevated in the PFC, while Cer(m18:1/18:2) and Cer(d17:1/18:0) were elevated in the hippocampus [18]. Conversely, some studies suggest that certain ceramides, including Cer(34:1), Cer(34:2), Cer(35:1), Cer(36:1), Cer(38:1), Cer(38:4), Cer(40:1), Cer(40:2), Cer(41:1), Cer(41:2), and Cer(42:2), consistently decrease in response to stress [19,20].

Experimental evidence also links ceramides to depression. For example, mice infused with Cer16 in the hippocampus exhibited depressive-like behaviors, while infusion of Cer8 or Cer20 did not induce similar effects [21]. Disruptions in sphingolipid metabolism have been further observed in rodent models of depression. Transgenic mice with overexpressed ASM showed elevated ceramide levels in the hippocampus, which correlated with increased depression severity [22]. Thus, elevated ceramide levels are associated with the onset and severity of depression, and reducing ceramide levels may help reverse depressive symptoms.

#### 2.1.2. Neuroprotective (SM)

Sphingomyelin (SM) is the most abundant sphingolipid in eukaryotic cells and constitutes a major component of the plasma membrane. The structural role of SM in cells is determined by its chemical structure as a phospholipid. Specifically, the hydrophilic head and hydrophobic tail of phospholipid molecules allow them to form a lipid bilayer structure. Naturally occurring SMs vary in fatty acid chain length (usually ranging from 16 to 24 carbons), as well as in their degree of saturation and hydrogenation. In summary, SM contains saturated fatty acids (SAFAs), monounsaturated fatty acids (MUFAs), and polyunsaturated fatty acids (PUFAs) [23].

Several studies have reported a decrease in SM levels following depression [14]. CUS results in reduced levels of various sphingomyelin species, including SM(16:0), SM(20:0), SM(22:0), SM(24:0), SM(26:0), dhSM(16:0), dhSM(16:1), dhSM(18:0), dhSM(18:1), dhSM(20:0), dhSM(22:0), dhSM(22:1), dhSM(24:0), dhSM(24:1), dhSM(26:0), dhSM(26:1), and dhSM(26:2) [16]. In the CVS model, sphingomyelin species with longer fatty acid chains, such as SM(d44:2), SM(d32:1), SM(d18:0/22:1), SM(d18:1/24:2), SM(d18:1/18:4), SM(d33:1), SM(d34:2), SM(t34:1), SM(d38:0), SM(d36:2), SM(d41:1), SM(d34:0), SM(d35:1), SM(d18:1/23:3), and SM(d37:1), were reduced in the PFC. In the hippocampus, levels of SM(d36:2), SM(d18:1/18:4), SM(d36:6), SM(d34:2), SM(d42:1), SM(d42:2), SM(d18:2/21:3), SM(d18:1/24:2), SM(d41:1), SM(d14:0/24:1), and SM(d36:1) also decreased [18].

Some studies, however, report no changes or increased SM levels after lipidomics analysis. For instance, after chronic CORT administration, the levels of dhSM16:1, dhSM20:0, dhSM20:1, dhSM22:1, dhSM26:0, dhSM26:1, and dhSM26:2 were increased in the ventral hippocampus [17]. The levels of SM, such as SM(d14:0/22:2), SM(d14:0/22:1), and SM(d18:1/18:0), were elevated after chronic unpredictable mild stress (CUMS) [20]. Also, no significant differences in SM content were found between control and chronic stress groups after CUS [24].

The precise mechanism by which SM influences depression remains unclear but is likely related to several factors, including the ceramide system, neuroplasticity, inflammatory processes, and cell membrane stability. The ASM-mediated breakdown of SM to ceramide and phosphorylcholine links SM metabolism to various physiological processes relevant to depression. Modulating SM metabolism may thus offer potential therapeutic effects. SM metabolites produced by sphingomyelinase, such as ceramide and sphingosine, are associated with pro-inflammatory cytokines and are considered therapeutic targets in the inflammatory processes linked to depression. Disruptions in SM metabolism may compromise cell membrane stability, which in turn can affect cellular function. Such alterations in membrane integrity may contribute to the onset or progression of depression. For example, some studies have shown that alterations in the SM metabolism pathway are implicated in depression, potentially decreasing cell membrane stability and triggering a cascade of pathophysiological changes [25].

#### 2.1.3. Neuroprotective (Sphingosine)

Recent studies have highlighted the critical role of 1-phosphosphingosine in brain health [26]. Several investigations have linked 1-phosphosphingosine to various neurological and psychiatric disorders, including Alzheimer’s disease, depression, and anxiety [27].

Sphingosine, a key intermediate in the sphingolipid metabolic pathway, connects ceramides and 1-phosphosphingosine. Ceramidase hydrolyzes ceramides into sphingosine, which is subsequently phosphorylated to form 1-phosphosphingosine. Elevated sphingosine levels in the PFC correlate with reduced depression-like behaviors and improved cognitive function. In contrast, the CUMS group showed a significant reduction in sphingosine levels in the PFC, indicating disrupted sphingolipid metabolism [28].

In addition, the sphingosine analogue fingolimod (FTY720) has been found to have significant neuroprotective effects on the CNS. It not only reduces neuronal damage and oxidative stress but also enhances cognitive function and alleviates depression-like behavior in rats. Additionally, FTY720 protects rat hippocampal neurons from CUMS-induced oxidative and inflammatory damage. It also mitigates depression-like symptoms by inhibiting the NLRP3 inflammasome in resident microglia, suggesting its potential as a promising neuroprotective agent in the treatment of depression [29].

**Figure 2:**
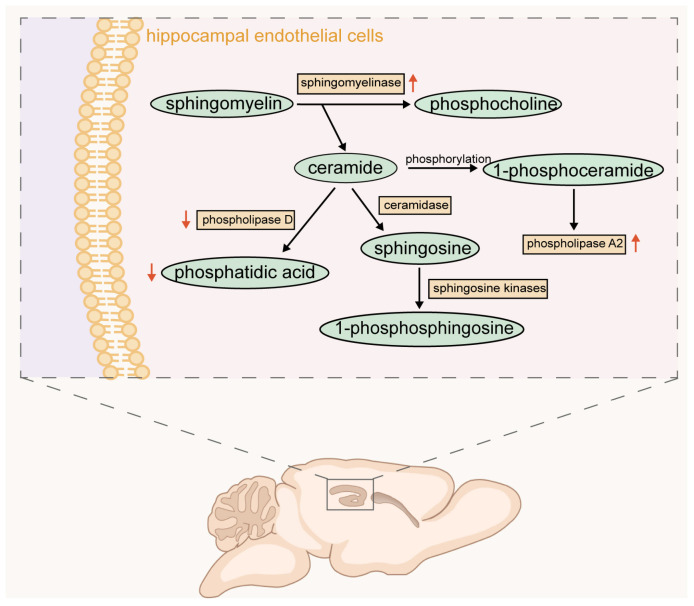
Hippocampal endothelial cell-related lipid transition. The acid sphingomyelinase–ceramide pathway. In hippocampal endothelial cells, sphingomyelin is broken down into phosphatidylcholine and ceramide via sphingomyelinase. Ceramide can be converted into phosphatidic acid via phospholipase D, or sphingosine via ceramidase, and further converted into 1-phosphosphingosine. Ceramide can also be phosphorylated into 1-phosphoceramide and then affect the activity of phospholipase A2. The red arrow represents the general change trend after depression: ⬆ represents activation/rise, ⬇ represents suppression/decrease.

In summary, sphingolipids play crucial roles in central nervous system function and pathology. Ceramides are linked to neurotoxic effects, while sphingomyelins and sphingosine exhibit neuroprotective properties by promoting the stability of cell membranes, thus supporting cellular function. The acid sphingomyelinase–ceramide pathway effectively illustrates the interconversions of these lipids (Figure 2). Modulating these lipids offers promising therapeutic strategies for conditions such as depression.

### 2.2. Glycerophospholipids, GPs

Phospholipids, particularly glycerophospholipids, are essential components of neuronal membranes and brain phospholipids. They play a critical role in regulating synaptic function. These phospholipids can be classified into various subtypes based on differences in their phospholipid glycerol backbones and polar head groups, including phosphocholine (PC), phosphatidylethanolamine (PE), phosphatidylserine (PS), phosphatidic acid (PA), and phosphatidylinositol (PI).

#### 2.2.1. Neurotoxic (PC, PE)

PC is the most abundant glycerophospholipid in eukaryotic cell membranes, comprising 40–50% of the cell membrane content across organisms. It plays a pivotal role in maintaining cell membrane architecture. Elevated PC levels in the brain can reduce acetylcholine release, potentially impairing synaptic plasticity [30]. PE, which serves as a precursor for PC biosynthesis, also influences membrane topology, thus promoting membrane activation.

Changes in PE and PC levels are closely linked to anxiety and depressive behaviors in rats with MDD. Specifically, in the PFC, PE levels are reduced, while both PE and PC accumulate in the hippocampus, worsening symptoms of despair and anxiety. In contrast, a study on late-onset depression (LOD) rats revealed differing trends in glycerophospholipid metabolism. In this case, the accumulation of PE and PC in the frontal lobe alleviated anxiety and depressive behaviors in LOD rats [31].

In the hippocampus, levels of specific PCs and PEs increased in rats injected with lipopolysaccharide (LPS), including PC(15:0/16:0), PC(16:0/16:0), PE(15:0/22:6), and PE(16:0/20:5) [32]. Similarly, in rats exposed to CUS for four weeks, levels of PC(36:3), PC(38:5), PC(38:2), PC(40:2p), PC(40:2), PC(14:0/20:4), PC(18:0p/18:1), PE(34:1e), PE(18:1p/18:1), PE(18:0p/18:1), PE(18:0/20:4), PE(16:0/20:4), PE(18:0/18:2), PE(20:1/18:1), and PE(22:5/18:2) were elevated [19]. In contrast, mice subjected to CVS for 21 days exhibited reduced levels of PE(34:0p), PC(36:4e), PC(38:7), PC(38:5), PC(40:7), PC(36:4), PC(18:1/24:0), PC(31:0/11:4), PC(8:1e/11:3), PC(26:1/12:4), PC(18:0/20:2), PC(16:2e/22:1), PC(20:2/20:3), and PC(14:0/20:4) [18]. Long-term corticosterone (CORT) exposure in the ventral hippocampus led to increased levels of PE(32:1), PE(34:2), PC(40:7), PC(42:6), and PC(42:7), while PC(38:0), PC(38:1), PE(36:0), PE(36:1), PE(38:0), PE(38:1), and PE(38:2) were significantly reduced [17]. In rats with post-stroke depression (PSD), sucrose preference positively correlated with hippocampal concentrations of PC(16:0/22:4), PC(16:0p/22:6), PC(20:1/20:4), PC(33:0), PE(18:0p/24:1), PE(39:1p), PE(40:0e), and PE(51:8). Additionally, immobility time negatively correlated with PC(20:1/20:4) levels in the hippocampus [33]. In adult rats subjected to chronic stress, PE species levels significantly declined in the PFC [16], along with alterations in PC concentrations [19]. Similarly, in mice exposed to CVS for 21 days, levels of PC(6:0/12:4), PC(36:4), PC(18:1/18:1), PC(25:0/11:1), PC(20:1/18:1), PC(34:1e), and PC(34:0e) decreased, whereas PE(16:0/20:4), PE(9:0/10:1), PE(20:1p/22:4), PE(13:0/10:4), PE(9:0/12:4), PE(11:0/10:1), and PE(18:1e/20:4) increased [18].

Depressed children exhibited significantly lower levels of phosphocholine and glycero-3-phosphocholine in the right anterior cingulate cortex compared to controls [34]. In elderly patients with depression, glycero-3-phosphocholine levels were higher in white matter than in gray matter, while glycero-3-phosphoethanolamine concentrations were significantly elevated in white matter compared to controls [35].

Synthesis of the phospholipids also occurs mainly in the ER [36]. Enzymes such as Pld2 and Ptdss1 play essential roles in glycerophospholipid metabolism. Pld2 catalyzes the hydrolysis of PE or PC, whereas Ptdss1 facilitates the conversion of PE or PC into phosphatidylserine [37]. Therefore, reductions in Pld2 and Ptdss1 may contribute to altered glycerophospholipid metabolism [24].

#### 2.2.2. Neuroprotective (CL)

Cardiolipin (CL) is a distinct dimeric phospholipid located in the inner mitochondrial membrane, where it is essential for optimal mitochondrial function. Due to the preferential generation of reactive oxygen species (ROS) within mitochondria, CL is more vulnerable to oxidation than other phospholipids [38]. During cell death, the increase in ROS and the loss of CL form a cycle of CL peroxidation [39]. Research has shown that CL oxidation, along with a decrease in CL content, correlates with neuronal loss or dysfunction induced by chronic stress. These alterations resemble those observed in neurodegenerative diseases, highlighting the pathophysiological processes associated with depression [40]. Mice exposed to stress stimuli exhibited an 80% increase in catalase activity compared to controls. Additionally, brain superoxide dismutase (SOD) activity differed significantly, and the GSH/GSSG ratio decreased by 44% [24].

Chronic stress leads to a notable decrease in various cardiolipin classes. For example, a significant reduction in 50 lipid metabolites, primarily cardiolipins, was detected in the hippocampus of rats with post-stroke depression, 4 weeks post-stroke. Specific reductions included CL(16:0/16:1/18:1/20:4) and others [33]. Similarly, in mice exposed to CUS for 21 days, reductions in key CL species such as CL(18:2/18:2/18:2/18:2) and others were observed [24]. Other studies have also reported decreases in CL species, such as CL(87:5) and CL(80:12), particularly in the hippocampus of mice subjected to CVS for 21 days, compared to controls [18].

#### 2.2.3. Neurotoxic (PI)

PIs are a widely distributed class of phospholipids involved in secretory processes and intercellular signaling. Their metabolism plays a critical role in signal transduction pathways regulated by various hormones, neurotransmitters, and growth factors. PI classes also serve as precursors for key signaling molecules that govern cell growth, proliferation, and apoptosis [41].

Recent studies have reported conflicting findings regarding PI alterations following CUS. One study observed a significant decrease in PI levels in the PFC of mice subjected to 21 days of CUS [24], while another reported reduced PI levels in the PFC after chronic stress [19]. However, other studies found increased PI levels in the hippocampus following four weeks of CUS in rats [16]. Specifically, PI(16:0/20:5), PI(18:1/22:6), and PI(10:0/20:4), among others, were elevated in the PFC of mice exposed to CVS compared to controls [18]. Similarly, PI(16:0/20:3), PI(16:0/20:4), and PI(18:0/20:3) increased in the hippocampus of LPS-induced rats [32]. Exposure to CORT for four weeks also elevated PI(36:3), PI(38:6), and PI(40:5) in the ventral hippocampus of rats, while PI(34:0), PI(34:1), PI(36:1), and PI(36:2) increased in both the dorsal and ventral hippocampus [17]. Overall, most studies indicate a general increase in PI levels under chronic stress conditions.

Phosphatidylinositol synthesis occurs in the ER with the participation of the CDP-diacylglycerol-inositol 3-phosphatidyl transferase (PIS) enzyme. Interconversion between various phosphoinositides, mediated by lipid kinases and phosphatases, occurs beyond the ER [36]. PIs can be phosphorylated into various phosphoinositides (PIPs), each with distinct functions depending on the phosphorylation site in the inositol head group. These molecules are crucial for initiating signaling cascades and play a central role in cell metabolism and apoptosis [42]. While the direct relationship between PIPs and depression remains unclear, it is hypothesized that PIPs may influence neurotransmitter levels by modulating their synthesis, release, or reuptake. Specifically, PIPs can promote or inhibit the transporter function of certain neurotransmitters, thus affecting their concentration in the synaptic cleft [43,44]. As a critical component of the inner cell membrane, PIPs are involved in regulating vesicle release and recovery, which significantly impacts synaptic plasticity. Abnormal synaptic plasticity can impair neuronal communication and potentially lead to depressive symptoms [45].

#### 2.2.4. Neuroprotective (PA)

PA, a minor yet essential class of glycerophospholipids, serves as a key intermediate in the synthesis of membrane and storage lipids. PA plays a critical role in various cellular and physiological processes across eukaryotes, from microbes to mammals and higher plants. PA levels are highly dynamic, responding to multiple stimuli and enzymatic reactions. Its unique physicochemical properties allow it to influence membrane structure and dynamics and interact with numerous proteins, making it crucial for cellular signaling. PA has emerged as a significant lipid mediator, modulating diverse signaling and cellular processes through mechanisms such as membrane tethering, conformational changes, enzymatic activity regulation, and vesicular trafficking [46].

Studies have shown that individuals with depression-like symptoms exhibit significantly increased synthesis and reduced degradation of PA in the amygdala (AMY). This finding correlates with elevated PA levels in the depression-like AMY, as measured by mass spectrometry [47]. Additionally, researchers observed a decrease in PA(48:4) levels in the hippocampus of the CVS group compared to controls [18]. In rats exposed to CORT, PA(32:0) and PA(34:2) levels increased in the dorsal hippocampus, while PA(38:1) and PA(38:2) levels decreased. Furthermore, PA(34:2) levels increased in the ventral hippocampus [17]. Although data remain limited, several mechanisms suggest that PA has neuroprotective effects. For example, PA is involved in the sphingomyelinase–ceramide pathway and regulates autophagy by influencing ganglioside (GM1) levels. PA may also affect depression development through multiple pathways, including regulating autophagy in neurons both in vitro and in vivo. Additionally, PA can bind to and regulate the activity of phosphotyrosine phosphatase 1B (PTP1B), which influences the tyrosine phosphorylation of various cellular proteins. Deficient PA leads to increased PTP1B activity, resulting in reduced tyrosine phosphorylation of TrkB and other proteins. These findings offer new insights into the pathophysiological mechanisms of depression and may identify potential targets for novel therapeutic strategies [48].

#### 2.2.5. Neuroprotective (PS)

PS is an acidic phospholipid crucial for brain neuronal membranes. Although it makes up only a small percentage of the phospholipids in biological membranes, PS accounts for approximately 13% to 15% of the total phospholipids in the human cerebral cortex [49,50]. Its presence in neural membranes is particularly important for the activation of protein kinase C (PKC), whose activity typically declines with age. Therefore, reduced PS levels in the brain may be linked to cognitive decline and impairment [51]. Supplementing PS helps sustain neurotransmitter-producing neurons and the production of acetylcholine, potentially preventing cognitive deficits. Chronic PS administration has been shown to reverse dendritic spine loss in the hippocampus of aged rats [52]. This effect on neuronal connectivity may contribute to PS’s antidepressant properties. Additionally, PS has been reported to restore dopamine release [53].

Recent lipidomic analyses have shown increased PS levels under stress. In rats subjected to daily CORT injections, PS(34:1), PS(36:2), PS(38:3), PS(40:6), and PS(42:5) levels increased in the ventral hippocampus, while PS(32:1) levels rose in both the dorsal and the ventral hippocampus [17]. Similarly, PS(16:0/18:1), PS(16:0/22:6), and PS(18:0/20:3) increased in the hippocampus of rats injected with LPS [32]. PS(39:0) levels increased in the hippocampus, and PS(18:1/18:1) levels increased in the PFC [19]. In contrast, PS(36:1), PS(36:2), PS(38:4), PS(40:4), PS(40:6), PS(18:0/20:4), and PS(46:2) decreased in mice after CUMS for 4 weeks [20]. These findings highlight the complex role of PS, which may be influenced by various stress conditions and experimental settings. It remains unclear whether the body produces compensatory PS in a depressed state.

### 2.3. Fatty Acyls, FA

#### 2.3.1. PUFA

Nerve tissue is rich in PUFAs, which are vital for proper neuronal function, including neurotransmission. Brain lipids predominantly contain long-chain omega-3 and omega-6 PUFAs, which modulate a variety of physiological processes, such as neuroinflammation, neurotransmission, and neuronal survival. These essential fatty acids cannot be synthesized by the body and must be obtained through the diet [54].

Docosahexaenoic acid (DHA) and eicosapentaenoic acid (EPA) are synthesized from α-linolenic acid (18:3, n-3) and γ-linolenic acid (GLA), along with arachidonic acid (ARA), derived from linoleic acid (20:4, n-6). These PUFAs play essential roles in maintaining membrane fluidity, lipid peroxidation, eicosanoid synthesis, receptor and channel function, and gene expression. Arachidonic acid, the most prominent n-6 PUFA in humans, has been extensively studied in the context of depression and other psychiatric disorders. This is due to its conversion by cyclooxygenase (COX) enzymes into biologically active compounds that participate in both physiological responses and pathological processes [55]. Studies have linked elevated ARA levels with neuroinflammation in patients with MDD [56]. Additionally, research suggests that ARA may increase CORT secretion, contributing to anxiety-like behavior in animals [57].

Depression has been shown to disrupt lipid synthesis regulation. DHA levels decrease in the hippocampus of MDD patients [58]. Chronic mild stress (CMS) reduces the content of docosapentaenoic acid (DPA) in the PFC, hippocampus, and striatum [59]. Exposure to CVS notably alters the fatty acyl chain profiles of lipids in both the hippocampus and PFC. Specifically, in the hippocampus, the levels of long-chain fatty acyls with fewer than 17 carbon atoms (17C) and 23 carbon atoms (23C) increase, along with elevated levels of saturated fatty acyls. In contrast, long-chain fatty acyls with 31 carbon atoms (31C) decrease. In the PFC, long-chain fatty acyls with fewer than 16 carbon atoms (16C) and 23 carbon atoms (23C) increase, while fatty acyls with 20 carbon atoms (20C), 21 carbon atoms (21C), 30 carbon atoms (30C), and 35 carbon atoms (35C) decrease. Additionally, polyunsaturated fatty acyls containing four double bonds also decrease in depression-like mice [18,19].

Dietary supplementation with n-3 PUFAs significantly increases DHA levels in the PFC and hippocampus while reducing DPA levels, without causing significant behavioral changes. In stressed animals, n-3 PUFA supplementation elevates tissue serotonin (5-HT) levels in the PFC and hippocampus, but not in the striatum. This suggests that the protective effects of n-3 PUFAs may be mediated by enhancing 5-HT activity specifically in the PFC and hippocampus. Furthermore, a DHA-depleted diet reduces DHA content in the brain in a region-specific manner, with the pituitary gland, cortex, hippocampus, cerebellum, and striatum exhibiting the most significant reductions [58]. Additionally, brain lipids are highly susceptible to oxidative stress, which induces lipid peroxidation under inflammatory conditions. During these conditions, PUFAs are converted into pro-inflammatory and anti-inflammatory oxylipins that contribute to both the promotion and resolution of inflammation [60].

Several randomized clinical trials have shown that n-3 PUFA supplementation improves clinical depression scores in adults, primarily due to the effects of EPA [61]. EPA has demonstrated antidepressant effects, particularly when combined with DHA [62]. Both DHA and EPA, whether used alone or in combination, may reduce the incidence of inflammation mediated by eicosanoids. They can decrease the production of pro-inflammatory cytokines such as tumor necrosis factor (TNF)-α, interleukin (IL)-1β, IL-2, and IL-6, which are influenced by eicosanoid release and are associated with depression [63]. Additionally, DHA and EPA can reduce inflammation via their precursor, arachidonic acid. By incorporating arachidonic acid into membrane-based phospholipids, DHA and EPA decrease both cellular and plasma arachidonic acid concentrations. Furthermore, EPA, but not DHA, may reduce arachidonic acid production by inhibiting delta-5-desaturase activity. Within the cyclooxygenase enzyme system, EPA competes with arachidonic acid for binding to phospholipase A2 (PLA2), thereby blocking the synthesis of pro-inflammatory eicosanoids such as prostaglandins, thromboxanes, and leukotrienes from arachidonic acid [64].

In the brain, DHA and ARA are present at concentrations around 10,000 nmol/g, whereas EPA typically has concentrations below 250 nmol/g in both humans and rodents [65]. EPA enters the brain at a rate similar to DHA, but its concentrations are 250–300 times lower due to rapid metabolism after crossing the blood–brain barrier. Although EPA does not accumulate in the brain, it is found in microglial cells, where its levels are at least twice those of DHA [66]. Homeostatic mechanisms regulate the esterification of EPA into phospholipids, which are essential for cell signaling [67]. Chronic stress increases the number of microglial cells, alters their morphology, and activates inflammatory markers, including inflammasomes, pro-inflammatory cytokines, and inflammatory signaling pathways across various brain regions. This stress-induced inflammation is often associated with depressive-like behaviors, such as anhedonia, reduced activity, social withdrawal, despair, and anxiety [68]. EPA and its bioactive metabolites are known for their immunomodulatory effects in peripheral immune cells and may exert direct regulatory actions on neuroinflammation through esterification and subsequent metabolism within microglia. Several preclinical studies have demonstrated that EPA provides neuroprotective and anti-inflammatory benefits in the brain [69].

#### 2.3.2. Neuroprotective (eCBs)

Endocannabinoids (eCBs) mainly include Anandamide (AEA) and 2-arachidonic acid glycerol (2-AG). The most common route of 2-AG synthesis involves the production of 2-AG from diacylglycerol (DAG) precursors by the action of 2-DAG lipases. The main pathway for the synthesis of AEA initially involves the synthesis of N-acylphosphatidylethanolamine (NAPE) from lipid membrane PE and PC by the calcium-sensitive enzyme N-acyl-transferase (NAT) [70].

AEA and 2-AG exert their effects primarily by binding to CB1 (type-1 cannabinoid receptor) and CB2 (type-2 cannabinoid receptor) receptors. In depression models, AEA and 2-AG levels are often reduced, leading to diminished CB1 receptor signaling. This suppression inhibits neurotransmitter release at presynaptic terminals, resulting in hyperactivation of the hypothalamic–pituitary–adrenal (HPA) axis and decreased hippocampal neurogenesis [71].

Despite its relatively low concentration in the central nervous system, AEA-mediated long-term depression is functionally relevant in multiple brain regions, including the dentate gyrus, amygdala, striatum, and nucleus accumbens. However, this effect does not appear to involve direct modulation of CB1 receptor activity or expression. Instead, it occurs through transient receptor potential vanilloid 1 (TRPV1) activation while inhibiting 2-AG-1 activity, potentially through glutathione system regulation [70].

### 2.4. Sterol Lipids, STs

#### 2.4.1. Neuroprotective (Cholesterol)

Cholesterol (CHOL) is a critical component of cell membranes, playing a pivotal role in regulating signaling molecules, modulating receptor activity, and serving as a precursor for steroid hormones. Increasing evidence suggests that CHOL has a wide range of functions and may influence various physiological processes. Studies in rodents indicate that peripheral and brain CHOL are independently regulated. In the CNS, CHOL homeostasis is maintained through de novo synthesis, membrane transport systems, or specific ligands, with excess CHOL being converted into 24S-hydroxycholesterol (24S-OH-Chol) and subsequently excreted into the systemic circulation [72]. Since CHOL cannot cross the blood–brain barrier, it is primarily synthesized in situ within the brain, with minimal transfer from plasma to the brain [73]. Most of the brain CHOL is located within myelin, with significant portions also found in the plasma membranes of neurons and glial cells. Given the slow turnover of myelin, CHOL within the myelin sheath is essentially immobilized. Researchers have suggested that the reduced CHOL levels observed in MDD are not the result of an overall loss of cortical myelin [74].

In individuals with MDD, total CHOL levels and low-density lipoprotein cholesterol (LDL-C) are reduced [75]. Specifically, CHOL levels in the visual association cortex are 13% lower compared to controls, with a significant decrease also observed in the cerebral cortex. However, levels of CHOL precursors such as lanosterol, lathosterol, desmosterol, and plant sterols like camposterol and sitosterol, as well as the CHOL metabolite 24S-OH-Chol, remain unchanged [74,76].

Recent research suggests that CHOL plays a crucial role in the development, function, and stability of synapses [77]. Glial cells secrete CHOL particles, which are then taken up by neurons through a receptor-mediated process. The transporter ABCA1 is essential for the transfer of CHOL from astrocytes to neurons. Downregulation of ABCA1 in neurons reduces CHOL efflux, while its upregulation increases lipid release [78]. These CHOL particles are subsequently internalized by neurons, supporting synapse formation and the maintenance of synaptic connections [79]. Recent evidence shows that when both glial cells and neuronal absorption decrease, ABCA1 levels increase, suggesting enhanced CHOL efflux. This alteration indicates disrupted CHOL homeostasis in the hippocampus, with reduced CHOL availability for synapses. Increased CHOL efflux combined with decreased reuptake promotes CHOL imbalance, impairs synaptic plasticity, and contributes to cognitive deficits. Based on these findings, researchers hypothesize that CUMS affects synaptic function and damages synaptic plasticity by disrupting CHOL homeostasis [30].

#### 2.4.2. BA

The brain is rich in CHOL, which serves as a precursor for bile acids (BAs). Although primary BAs, such as chenodeoxycholic acid (CDCA), can be synthesized in the brain, there is no evidence to support the synthesis of secondary BAs within the brain. This suggests that the primary source of brain BAs is the systemic circulation [80]. BAs enter the brain via BA transporters [81] and contribute to the pathogenesis of various neuropsychiatric disorders [82]. CDCA and deoxycholic acid (DCA) influence depression by altering the permeability of the blood–brain barrier, disrupting tight junctions in rat brain microvascular endothelial cells (RBMECs) [83] and interacting with various metabolites and enzymes to mediate neurological and inflammatory responses [84].

Recent studies suggest that BAs play both beneficial and detrimental roles in the CNS. Several BAs, including ursodeoxycholic acid (UDCA), taurochenodeoxycholic acid (TCA), glycochenodeoxycholic acid (GCDCA), and tauroursodeoxycholic acid (TUDCA), have been shown to mediate apoptosis in neuronal cells [85]. In patients with MDD, GCDCA and glycocholic acid (GCA) levels are significantly reduced, and multiple bile acids (GCDCA, GCA, GDCA, GUDCA) are negatively correlated with depression scores [86].

TUDCA is widely studied for its protective effects in brain diseases, demonstrating anti-apoptotic, anti-inflammatory, and antioxidant properties [87]. TUDCA treatment reversed the LPS-induced increases in malondialdehyde (MDA) and nitrite levels, as well as the reduction in glutathione (GSH) levels in both the hippocampus and prefrontal cortex. These findings suggest that TUDCA may help alleviate depressive behaviors [88]. In patients with MDD, a distinctive pattern of decreased CDCA levels, accompanied by an increase in lithocholic acid (LCA), holds particular pathophysiological significance. LCA is the most potent ligand for the Takeda G-protein-coupled bile acid receptor and a strong activator of the pregnane X receptor and the vitamin D receptor [89]. By binding to and activating these receptors, bile acids regulate their synthesis, conjugation, transport, and detoxification, as well as lipid, glucose, and energy homeostasis [82]. UDCA, the 7β isomer of CDCA, exhibits anti-apoptotic, anti-inflammatory, antioxidant, and neuroprotective effects in various neurodegenerative disease models [90]. Additionally, dehydrolithocholic acid, a major metabolite of LCA, is strongly negatively correlated with anxiety levels in MDD patients [91].

BA concentrations appear to have a stronger influence on anxiety symptoms than on depressive symptoms. Several secondary BA concentrations, as well as the ratios of secondary to primary BAs, differ significantly between MDD participants with low and high anxiety levels, regardless of depression severity. Notably, secondary BAs such as LCA and its derivatives, including 7-keto-LCA, isoLCA, alloLCA, and 12-ketoLCA, correlate with increased anxiety severity [91].

In depression, reduced cholesterol levels are linked to synaptic dysfunction and cognitive decline. This imbalance in cholesterol homeostasis primarily affects the synaptic cholesterol supply and ABCA1-mediated cholesterol transport. Bile acids, cholesterol metabolites, exert dual roles in the CNS, mediating both apoptotic and anti-apoptotic, anti-inflammatory, and antioxidant effects in neuronal cells. Altered bile acid levels are strongly associated with the symptoms of both depression and anxiety.

### 2.5. Glycerolipids, GLs

Diacylglycerol (DG) is a crucial membrane signaling lipid in the brain. It is primarily generated through the hydrolysis of phosphatidylinositol-4,5-bisphosphate by phospholipase C (PLC). Diacylglycerol kinases (DGKs) terminate DG signaling by converting it into PA, a key lipid second messenger. Through this process, DGKs regulate intracellular lipid signaling by both deactivating DG and generating PA [92].

The current study demonstrated that CUS elevated glycerolipid levels, including DG, triglycerides (TGs), and monoglycerides (MGs) in the hippocampus, as well as DG in the PFC. Lipidomic analysis revealed a significant increase in DG(18:0/20:4) concentrations in both the hippocampus and PFC [19]. Additionally, TG(16:0/16:0/20:4), TG(16:1/14:0/16:1), and MG(20:4) levels were elevated in the hippocampus of rats exhibiting depression-like behavior [32]. In the ventral hippocampus, CORT exposure increased the levels of DG(30:1/14:0), DG(32:1/16:0), and DG(32:2/16:1), among others. In the dorsal hippocampus, TG(52:5/18:1), TG(54:5/18:1), TG(54:6/18:1), and TG(56:9/20:4) levels were elevated. Notably, three DGs and nine TGs exhibited increased levels in both hippocampal regions [17]. Similarly, macaques with depression-like symptoms showed altered levels of 1,2-diacylglycerides, particularly those with carbon chain lengths of 34C, 36C, and 43C in the PFC and 38C in the AMY and hippocampus. Most differentially regulated DGs ranged from 34C to 40C. Compared with controls, DG unsaturation increased significantly in the PFC, specifically, in species with 2, 3, and 10 double bonds, while no significant changes in DG unsaturation were observed in the AMY or hippocampus [47].

Clinical studies investigating the relationship between glycerolipid (GL) levels and depression have yielded inconsistent results [7]. Some studies report positive correlations between sucrose preference and TG levels [33], while others observe reduced TG levels in the CUMS model [75]. Additionally, studies have found decreased levels of MG(32:0, 34:0) in the PFC under the CVS model, as well as decreased levels of DG(18:1/22:0), DG(18:1/24:0), phosphatidylglycerol (PG) (42:0), DG(18:1/24:1), and DG(24:1/20:4) in the hippocampus under the same model [18]. Other research also indicates a reduction in DG(16:0/20:4), DG(18:0/20:4), and several TG species (e.g., TG(54:2/18:0), TG(56:3/18:1), TG(56:8/20:4)) in the dorsal hippocampus [17,20].

Elevated TG levels may contribute to cerebrovascular lesions by impairing blood vessel elasticity and lumen size, resulting in altered cerebral hemodynamics [93]. These changes may be linked to the development of depression. Additionally, abnormal triglyceride metabolism may play a role in depression pathogenesis by affecting neurotransmitter regulation, neuroendocrine function, neuroimmune responses, and neuroplasticity. However, the exact mechanisms underlying these effects remain to be fully elucidated.

Furthermore, the lipid classification of prenol lipids, saccharolipids, and polyketides in depression remains relatively underexplored. Prenol lipids play critical roles in cell signaling and antioxidant defense. For instance, coenzyme Q, a key component of mitochondrial function, is involved in aging and neurodegenerative diseases such as Alzheimer’s and Parkinson’s diseases, as well as in degenerative muscle diseases [94]. Saccharolipids, a class of glycolipids, are widely distributed on animal cell membranes and regulate cell recognition, adhesion, proliferation, and apoptosis. They may also be implicated in hyperlipidemia [95]. Additionally, certain polyketides have shown protective effects against Alzheimer’s disease [96].

## 3. Conclusions and Perspective

A growing body of evidence highlights the increasing prevalence of MDD in global populations. Despite the central role of lipids in neurophysiology and pathology, their involvement in MDD has received relatively limited attention. In this review, we have synthesized current knowledge on brain lipid alterations to explore the impact of chronic stress on distinct brain regions (Table 1) and to elucidate the complex interactions between brain lipids and the pathophysiology of MDD. Lipids, particularly those integral to membrane structure and signaling, undergo significant alterations in MDD. These changes may contribute to the disease’s etiology by affecting neurotransmitter metabolism, energy metabolism, oxidative stress, and neuroinflammation (Table 2).

By categorizing brain lipids into neuroprotective and neurotoxic classes, we provide a framework for understanding their differential effects on depressive symptoms. Neuroprotective lipids, such as SM and certain PUFAs, seem to promote neuronal health and function. In contrast, neurotoxic lipids, such as ceramides, are associated with heightened inflammation, apoptosis, and oxidative stress—key features of MDD. However, due to variability in depression modeling methods, experimental durations, and animal models, lipidomics findings across different studies remain inconsistent. Therefore, the precise relationship between many lipids and the mechanisms underlying depression requires further clarification.

Several research directions warrant further investigation. First, developing lipid-based biomarkers for MDD could significantly improve both diagnosis and treatment monitoring. Biomarkers are crucial in the study of psychiatric disorders. For example, during the interictal period, abnormal neuronal activity around epileptic foci can be detected by fMRI, which can serve as a potential biomarker for the localization and assessment of epilepsy [97]. In Alzheimer’s disease (AD), hippocampal volume loss occurs early, and measuring hippocampal atrophy using high-resolution structural MRI scans can serve as a biomarker for both disease progression and diagnosis [98]. Similarly, changes in the composition and content of cerebrospinal fluid (CSF) proteins can reflect the state of brain diseases. For instance, β-amyloid (Aβ) and tau proteins in CSF are well-established biomarkers for AD [99], while inflammation-related proteins are elevated in the CSF of patients with central nervous system infections, such as bacterial meningitis [100]. Given these precedents, lipid changes in the brain could potentially serve as biomarkers for MDD, opening new opportunities for precise diagnosis and targeted treatment of the disorder.

Second, lipids such as ceramides, sphingolipids, and phosphatidic acids not only contribute to the structure and function of cell membranes, influencing neuronal and cellular activities, but also undergo interconversion through enzyme catalysis, forming a complex metabolic network. These lipid transformations may impact depression levels and manifest in various depressive states. Future research should focus on the interactions between these lipids, examining how they correlate with depression severity and how lipid metabolic networks change under different depressive conditions. This approach could provide a solid theoretical foundation for developing personalized treatments and novel drug therapies.

Third, several physiological processes, including glucose, lipid, and energy metabolism, are regulated by the circadian system. The synthesis, breakdown, and transport of lipids exhibit rhythmic fluctuations. Brain activity peaks during the day, requiring neurons to consume more energy. Currently, lipid catabolism may be elevated to provide the necessary energy to support neural activity. At night, when the brain is in a more restful state, lipid anabolism likely prevails, facilitating the repair and renewal of structures such as neuronal cell membranes. Additionally, hormones involved in lipid metabolism, such as insulin and glucagon, follow a circadian rhythm, influencing lipid turnover and indirectly regulating lipid synthesis. This cyclical regulation may help explain why depressive symptoms often worsen at night. Investigating nocturnal lipid fluctuations in individuals predisposed to depression could reveal whether lipid imbalances affect lipid synthesis or neurotransmission, and how these changes may relate to emotion-regulating brain regions.

Finally, techniques such as magnetic resonance spectroscopy may help assess whether recurrent depression correlates with more severe disturbances in brain lipid metabolism. Targeting lipid metabolism, particularly by enhancing neuroprotective lipids, could present novel therapeutic strategies for MDD. Moreover, understanding why lipidomic profiles differ across depression models and how lipid changes vary among brain regions remains crucial and warrants further research.

## Figures and Tables

**Table 1:** Lipids main changes in brain regions.

Lipid		Hippocampus	PFC	AMY	Cerebellum	Other Regions	Directionality of the Changes	References
SP	Cer	√	√	×	×		neurotoxic	[14,16,17,18,19,20,21,22]
	SM	√	√	/	/		neuroprotective	[14,16,17,18,20,24]
	Sphingosine	√	/	/	/		neuroprotective	[28]
GP	PC	√	√	/	/	anterior cingulate cortex, white matter	neurotoxic	[16,17,18,19,31,32,33,34,35]
	PE	√	√	/	/	white matter	neurotoxic	[16,17,18,19,31,32,33,34,35,36]
	CL	√	/	/	/		neuroprotective	[18,24,33]
	PI	√	√	/	/		neurotoxic	[16,17,18,19,24,32]
	PS	√	/	/	/		neuroprotective	[17,19,20,32]
	PA	√	√	√	/		neuroprotective	[16,17,46]
FA	DHA	√	√	√	√	pituitary gland		[54,55,56,57,58]
	EPA	√	√	/	/			[54,55,56,57,58]
	eCBs	/	/	√	/	dentate gyrus, striatum, nucleus accumbens	neuroprotective	[69,70]
ST	Cholesterol	√	√	/	/	visual association cortex	neuroprotective	[71,72,73]
	BA	√	√	/	/			[84,85,86,87,88,89,90]
GL		√	√	√	/			[7,17,18,19,20,32,33,46,74]

√ Changes mentioned in the article. × No change mentioned in the representative article. / not mentioned in the article.

**Table 2:** The mechanism by which brain lipids affect depression.

	Lipid	Mechanisms Influencing Depression
SP	Cer	acid sphingomyelinase–ceramide pathway; impair neuronal proliferation, maturation, and survival; enhance oxidative stress; pro-inflammatory; mediate the pro-inflammatory cytokines
	SM	acid sphingomyelinase–ceramide pathway, neuroplasticity, inflammatory processes, cell membrane stability, affect the normal function of cells
	Sphingosine	protect rat hippocampal neurons against oxidative and inflammatory damage by inhibiting the NLRP3 inflammasome in the resident microglia
GP	PC	higher brain levels of PC cause a decrease in acetylcholine release, may lead to the impairment of the brain synapses plasticity
	PE	influence the topology of cell membranes, thereby promoting membrane activation
	CL	an increase in catalase activity in brain mitochondrial membrane associated with neuronal loss or dysfunction
	PI	phosphorylated with formation of phosphorylated phosphoinositides, trigger signaling cascades, play a main role in cell metabolism and apoptotic cellular events, modulate neurotransmitter levels
	PS	activation protein kinase C, maintenance of the amount of the neurons that produce neurotransmitters, improve neurotransmission by glutamic acid
	PA	acid sphingomyelinase–ceramide pathway, regulation of autophagy by regulating ganglioside (GM1) levels, bind to and control the activity of PTP1B
PUFA	DHA, EPA	maintaining membrane fluidity, lipid peroxidation, eicosanoid synthesis, receptor and channel functioning, and gene expression; neuroprotective; anti-inflammatory
	ARA	correlated with neuroinflammation, cause an increase in the secretion of corticosterone
	lipid raft	reduce neurotransmitter signaling, lead to aberrant G-protein coupled receptor signaling
ST	Cholesterol	support synapse generation, maintain synaptic connections, affect synaptic plasticity
	BA	affect the permeability of the BBB by disrupting tight junctions of RBMECs, mediate neurological activity and inflammation
GL		affect cerebral hemodynamic changes, neurotransmitters, neuroendocrine, neuroimmune regulation, and neuroplasticity

Mechanism of change in lipids mentioned in the article.
